# Acknowledgement of manuscript reviewers, the underappreciated contributors

**DOI:** 10.1186/1743-7075-10-17

**Published:** 2013-02-20

**Authors:** Lucy Abel, Ahmed Bakillah, M Mahmood Hussain

**Affiliations:** 1 BioMed Central, 236 Gray's Inn Road, WC1X 8HB, London, UK; 2 Department of Cell Biology, SUNY Downstate Medical Center, 450 Clarkson Ave, 11203, Brooklyn, NY, USA

## Abstract

**Contributing reviewers:**

We and the Editorial Board acknowledge and thank all reviewers for their active participation and contribution during 2012. We greatly appreciate their dedication and behind the scenes contribution. It is largely due to their support and expertise that we have been able to publish high-standard manuscripts. We would also like to thank authors for choosing Nutrition & Metabolism and contributing their cherished work.

## 

Kadega Abdel Hameed

Egypt

Mobeen Abdollahi

Italy

Nilüfer Acar-Tek

Turkey

Khosrow Adeli

Canada

Soumyakanti Adhikari

India

Robert Adler

United States of America

Kolapo Ajuwon

United States of America

Mahmood Akhtar

United Kingdom

Hayder Al-Aubaidy

Australia

Jaume Almirall

Spain

Raj Amin

United States of America

Elitsa Ananieva-Stoyanova

United States of America

G. Harvey Anderson

Canada

Ramaroson Andriantsitohaina

France

Joshua Anthony

United States of America

Vasu Appanna

Canada

Ambika Ashraf

United States of America

Arne Astrup

Denmark

Bela Asztalos

United States of America

Lynn Htet Htet Aung

China

Salman Azhar

United States of America

Alfred Aziz

Canada

Alexander Bachmanov

United States of America

Zahra Bahadoran

Iran

Ahmed Bakillah

United States of America

Sanjay Kumar Banerjee

India

Tyler Barker

United States of America

Vicente Barrios

Spain

Elena Bartkiene

Lithuania

Jamie Baum

United States of America

Margaret Baumgarten

United States of America

Vic Ben-ezra

United States of America

Ina Bergheim

Germany

Shoma Berkemeyer

Germany

Bodil Bjorndal

Norway

William Blaner

United States of America

James Blumenthal

United States of America

Giuseppe Boccuzzi

Italy

Antonio Bonetti

Italy

Maria Halina Borawska

Poland

Sherazede Bouderbala

Algeria

Jeffrey Brault

United States of America

Leigh Breen

Canada

Despina Briana

Greece

Samantha Brooks

Sweden

Roland Buettner

Germany

Nicholas Burd

Netherlands

Craig Burkhart

United States of America

Mario Calomme

Belgium

Craig Canapari

United States of America

Patrice D. Cani

Belgium

Marly Cardoso

Brazil

J Carson

United States of America

Subrata Chakrabarti

Canada

Etienne Challet

France

Loanda Cheim

Brazil

Hsiao-Huei Chen

Canada

Dong Cheng

United States of America

Streamson Chua

United States of America

Christelle Chua

Singapore

Peter Clifton

Australia

Jeffrey Cohn

Australia

Kate Collison

Saudi Arabia

Roberto Conti

Italy

Marcia Cooper

Canada

Giuseppe Corona

Italy

Philippe Costet

France

Sheila Costford

Canada

Magna Cottini F. Passos

Brazil

Mario Cozzolino

Italy

Jim Daily

United States of America

Vincent Dalbo

Australia

Barbara Dao

Canada

Dominique Dardevet

France

Marcus Darrabie

United States of America

Nicholas Davidson

United States of America

Rini de Crom

Netherlands

Giovanni De Pergola

Italy

Andrea Deierlein

United States of America

Yves Deshaies

Canada

Madiha Dhibi

Tunisia

Mattia Di Nunzio

Italy

Ning Ding

United States of America

Alicia Doerflinger

United States of America

Sandra Dos Santos

United States of America

Daphna Dror

United States of America

Michael Dubick

United States of America

Pierre-Henri Ducluzeau

France

David Dyck

Canada

Mohamed El-Far

Canada

Yulia Elkina

Germany

Abdalla El-Mowafy

Egypt

Feraye Esen Gursel

Turkey

Joel Faintuch

Brazil

Jenifer Fenton

United States of America

John Fernstrom

United States of America

Judith Flanagan

Australia

Elisabet Forsum

Sweden

Jan Frank

Germany

Lynda Frassetto

United States of America

Asmaa Fritah

France

Gotthold Gäbel

Germany

Nicholas Gabler

United States of America

Mary C. Gannon

United States of America

Pablo M Garcia-Roves

Spain

Maude Gerbaix

France

Paramita Ghosh

United States of America

Vasileios Giapros

Greece

Javier Gisbert

Spain

Fernando Gomollón García

Spain

Juan Gormaz

Chile

László Góth

Hungary

Rejeanne Gougeon

Canada

Beau Greer

United States of America

Bart Groen

Netherlands

Kyle Hackney

United States of America

Mirko Hadzija

Croatia

Lena Håglin

Sweden

Tiruneh Hailemariam

United States of America

Ruijun Han

United States of America

Markolf Hanefeld

Germany

K.V.S. Hari Kumar

India

Robert Harris

United States of America

Fiona Harrison

United States of America

David Hauton

United Kingdom

Daniel Hayes

United States of America

Ashley Herda

United States of America

Ernesto Hernandez

United States of America

Erica Hinckson

New Zealand

Sandra Hirsch

Chile

Zhijun Hou

China

G Kees Hovingh

Netherlands

Miloslav Hronek

Czech Republic

Adela Hruby

United States of America

Olivier Huck

France

Ferenc Husveth

Hungary

Daniel Hwangt

United States of America

Shinichi Ikushiro

Japan

Jasminka Ilich

United States of America

Mustapha Umar Imam

Malaysia

Akihiro Inazu

Japan

Harvey Indyk

New Zealand

Sheila Innis

Canada

Jahangir Iqbal

United States of America

Ashley Simon Izzard

United Kingdom

David Jacobs

United States of America

Abhay Jagtap

India

Amanda Jiménez

Spain

Joby Josekutty

United States of America

Andrea Josse

Canada

Kim Kah Hwi

Malaysia

Enikoe Kallay

Austria

Amani Kallel

Tunisia

Santosh Katiyar

United States of America

Mamdouh Kedees

United States of America

Michael Keenan

United States of America

Darshan Kelley

United States of America

Philip Kerr

Australia

Tim Key

United Kingdom

Ji Yeon Kim

South Korea

Byung-Eun Kim

United States of America

Kyoung Kon Kim

South Korea

John Kirwan

United States of America

Mitchell Knutson

United States of America

Kaz Koba

Japan

S Eleonore

Koehler Netherlands

Suminori Kono

Japan

Rene Koopman

Australia

Jan Kopecky

Czech Republic

Zuzana Kovacova

Czech Republic

Herculina Salome Kruger

South Africa

Osman Kucuk

Turkey

Ujendra Kumar

Canada

Oran Kwon

South Korea

Steen Larsen

Denmark

Enette Larson-Meyer

United States of America

James Lattimer

United States of America

Fabienne Laugerette

France

Fulvio Lauretani

Italy

Martine Laville

France

Paul LeBlanc

Canada

Magalie Leduc

United States of America

Ok-Hwan Lee

South Korea

LeeCole Legette

United States of America

Tung Ming Leung

United States of America

Defa Li

China

Fabio Lima

Brazil

Julie Lin

United States of America

Pao-Hwa Lin

United States of America

Wenhua Ling

China

Barbara Lingwood

Australia

Ju-Chi Liu

Taiwan

Margarita Llera-Moya

United States of America

Liu Lu Shan

China

Fiona Lynch

United Kingdom

Zuzana Macek Jilkova

France

Meenalakshmi Mariappan

United States of America

Daniel Marks

United States of America

Cyrille Maugeais

Switzerland

Gaetan Mayer

Canada

Claire McEvoy

United Kingdom

Owen McGuinness

United States of America

Syed Musthapa Meeran

India

Rania Mekary

United States of America

Edward Melanson

United States of America

Jorge Mendoza

France

Christine Metzner

Germany

Nils Milman

Denmark

Parvin Mirmiran

Iran

Ali Moazzami

Sweden

Adam Moeser

United States of America

El-Jawed Mohamed

Kuwait

Sheba Mohan Kumar

United States of America

Anselmo Moriscot

Brazil

Margaret Morris

Australia

Fabio Mosca

Italy

Carmen Moura

Brazil

Naima Moustaid-Moussa

United States of America

Naofumi Mukaida

Japan

Manuel Muñoz

Spain

Kentaro Murakami

Japan

Vikkie Mustad

United States of America

Manar Nader

Egypt

Syed Nasar-Abbas

Australia

Julie-Anne Nazare

Canada

Takahiro Nemoto

Japan

Monika Neuhauser-Berthold

Germany

Ake Nilsson

Sweden

Katharina Nimptsch

United States of America

Wenze Niu

United States of America

Ethel Novelli

Brazil

Patricia Nunes

Netherlands

Rima Obeid

Germany

Yuichi Oishi

Japan

Lawrence Olatunji

Nigeria

Luqman Olayaki

Nigeria

Paulo Oliveira

Portugal

Johan Olsson

Sweden

Thaddeus Pace

United States of America

Lucia Pacifico

Italy

Mariona Palou

Spain

Jie Pan

China

Xiaoyue Pan

United States of America

Daniel Paredes-Sabja

Chile

Juyeon Park

South Korea

Phani Patra

United States of America

Bo Pei

United States of America

Michaela Pekarova

Czech Republic

Botond Penke

Hungary

Sandra Peters

Canada

Stephen Phinney

United States of America

Jingbo Pi

United States of America

Gustavo Duarte Pimentel

Brazil

Carlos Hermano J. Pinheiro

Brazil

Richard Planells

France

Graca Porto

Portugal

Nebojsa Potkonjak

Serbia

Parinaz Poursafa

Iran

Resia Pretorius

South Africa

Spencer Proctor

Canada

Joseph Prohaska

United States of America

Jeanine Prompers

Netherlands

Peter Pushparaj

United Kingdom

Eoin Quinlivan

United States of America

Mizanur Rahman

Bangladesh

Naga Rajan

India

Blake Rasmussen

United States of America

Suresh Rattan

Denmark

Raylene Reimer

Canada

Raquel Retana-Ugalde

Mexico

James Rippe

United States of America

Manfredi Rizzo

Italy

Denise Robertson

United Kingdom

Jacques Robidoux

United States of America

Viviane Rocha

Brazil

Edmond Rock

France

Olav Rooyackers

Sweden

Jean-Max Rouanet

France

Jan Rozman

Germany

Elaine Rush

New Zealand

Lekha Saha

India

Adolfo Saiardi

United Kingdom

Rocío Salceda

Mexico

Abdelbaset Saleh

Egypt

Silvia Savastano

Italy

Sonia G Sayago Ayerdi

Mexico

Herb Schellhorn

Canada

Mary Schooling

Hong Kong

Matthew Schubert

United States of America

Michael Scott

United States of America

Francisca Serra

Spain

Kartik Shankar

United States of America

Pengxiang She

United States of America

Oleg Sidorenkov

Norway

Rakesh Singh

United States of America

Brij Singh

United States of America

Som Nath Singh

India

Kshetrimayum Birla Singh

India

Jon Skorve

Norway

Adrian Slee

United Kingdom

Zeina Soayfane

United States of America

James Soh

United States of America

Rosa Solà

Spain

Manjunath Somannavar

India

Rai Ajit Srivastava

United States of America

Ken Stark

Canada

Christodoulos Stefanadis

Greece

Christine Stewart

United States of America

Keith Stokes

United Kingdom

Tatsuya Sueyoshi

United States of America

Papia Sultana

Bangladesh

Michael Swarbrick

Australia

Daniel Taber

United States of America

Maria Tabernero

Spain

Youcai Tang

United States of America

Simona Tecco

Italy

Daniel Teitelbaum

United States of America

John Thyfault

United States of America

Uwe Tietge

Netherlands

Aron Troen

Israel

Chi Tzong Cherng

Taiwan

Felipe Vadillo-Ortega

Mexico

Mohammadreza Vafa

Iran

Angela Valverde

Spain

Felix Van der Meer

Netherlands

Marta Van Loan

United States of America

Yvan Vandenplas

Belgium

Renée Ventura-Clapier

France

Anne-Claire vergnaud

United Kingdom

Carla Maria Vieira

Brazil

Nathalie Viguerie

France

Jeff Volek

United States of America

Chunlin Wang

China

Xiangbing Wang

United States of America

Zhao Wang

United States of America

Fei Wang

United States of America

Peter JM Weijs

Netherlands

Stephen Weinmann

United States of America

Edward Weiss

United States of America

Adam Whaley-Connell

United States of America

John White

United States of America

Katrina Williams

Australia

Pattanee Winichagoon

Thailand

Oliver Witard

United Kingdom

Richard Wood

United States of America

Shunjiang Xu

China

Yong Xu

United States of America

Hariom Yadav

India

Shigeru Yamamoto

Japan

Naomi Yasui

Japan

Zhong Ye

United States of America

Jingdong Yin

China

Chundong Yu

China

Kyeong Ho Yun

Korea, South

Khatijah Yusoff

Malaysia

Li Yuxiu

China

Vitali Zagorodnov

Singapore

Jun Zhang

United States of America

Weili Zhang

China

Xia Zhang

China

Hang Zhao

United States of America

Shuang-Xia Zhao

China

Dequan Zhou

United States of America

Robert Zoeller

United States of America

Antonio Zorzano

Spain

